# Systemic Integrative Mechanisms and Intervention Strategies in Exercise-Induced Skeletal Muscle Damage: Evidence from Animal, Clinical, and Multi-Omics Studies

**DOI:** 10.3390/ijms27052451

**Published:** 2026-03-06

**Authors:** Tianhang Peng, Zike Zhang, Ju Wei, Ni Ding, Wanyuan Liang, Xiuqi Tang

**Affiliations:** 1 School of Sports Science, Beijing Sport University, Haidian District, Beijing 100084, China; 2 College of Physical Education, Hunan Normal University, Changsha 410012, China; 3 School of English Studies, Tianjin Foreign Studies University, Tianjin 300204, China

**Keywords:** exercise-induced muscle damage, stress integration, oxidative stress, ferroptosis, satellite cells, multi-omics integration

## Abstract

Exercise-induced muscle damage (EIMD) has classically been attributed to localized mechanical disruption following eccentric contractions. Emerging evidence, however, indicates that EIMD represents a systems-level failure of stress integration within skeletal muscle rather than a purely mechanical lesion. Mechanical loading initiates disturbances in intracellular Ca^2+^ homeostasis, which interact with metabolic stress, redox imbalance, and immune activation to form self-reinforcing feedback loops. When compensatory capacity is exceeded, transient injury may shift toward maladaptive remodeling marked by mitochondrial dysfunction, ferroptosis, chronic inflammation, and impaired regeneration. Recent studies identify reactive oxygen species accumulation, iron-dependent lipid peroxidation, dysregulated energy sensing, and aberrant immune polarization as key molecular tipping points governing injury reversibility. Beyond their regenerative role, satellite cells act as integrators of metabolic history and epigenetic memory, linking repetitive injury to reduced muscle adaptability, age-related sarcopenia, and heightened metabolic disease risk. Here, we synthesize evidence from animal models, clinical studies, and multi-omics analyses to establish a systems biology framework for EIMD. We delineate the spatiotemporal interactions among mechanical, metabolic, oxidative, immune, and regenerative modules; identify regulatory nodes that determine adaptive repair versus pathological outcomes; and critically evaluate current nutritional, physical, pharmacological, and regenerative interventions from a mechanism-oriented perspective. Finally, we discuss how multi-omics, digital monitoring, and individualized rehabilitation may enable precision management of EIMD and advance understanding of muscle stress resilience and adaptive limits.

## 1. Introduction

Exercise-induced muscle damage (EIMD) refers to a spectrum of structural and functional alterations that occur in skeletal muscle when stress loads imposed by intense or novel exercise exceed physiological adaptive thresholds. These alterations include microtears of muscle fibers, compromised sarcolemmal integrity, edema formation, enhanced oxidative stress, and activation of inflammatory responses [1,2,3,4]. Traditionally, EIMD has been primarily attributed to localized mechanical damage induced by eccentric contractions, with characteristic pathological features such as sarcomere misalignment, Z-disk disruption, and myofibrillar disintegration [5,6,7,8,9]. However, advances in molecular biology and systems physiology have increasingly demonstrated that EIMD is not an isolated structural insult, but rather reflects a systemic breakdown of skeletal muscle homeostasis driven by impaired integration of multiple stress signals. Eccentric exercises, such as downhill running or resistance training, involve muscle active contraction accompanied by passive stretching, resulting in skeletal muscle fibers undergoing elevated active strain in a non-homogeneous mechanical environment. Classic experimental studies suggest that it is this excessive active strain, rather than the instantaneous force or stress levels themselves, that constitutes a key mechanical determinant in the initiation of EIMD [2,6,10]. During this process, locally “weaker” sarcomeres undergo excessive overstretching and rupture, leading to microdisruptions of the sarcolemma and transverse tubule system, thereby perturbing the finely tuned ionic homeostasis of skeletal muscle [8]. Clinical and experimental observations further reveal substantial inter-individual variability in the severity of muscle damage and recovery trajectories, even under comparable mechanical loading conditions [11,12,13,14]. This variability underscores that EIMD cannot be adequately explained as a purely mechanical event, but instead represents a systemic collapse of homeostatic regulation arising from dysregulated integration of mechanical, metabolic, oxidative, and immune stress signals.

EIMD exhibits a high prevalence in both athletic and general populations. Elite athletes undergoing high-intensity endurance or explosive training are particularly susceptible to cumulative damage when training loads fluctuate excessively or recovery is insufficient [11,15]. Similarly, untrained individuals exposed to unfamiliar exercise stimuli face elevated risk due to the absence of prior adaptive conditioning [11]. With the growing global participation in high-intensity physical activity, EIMD has emerged as a significant public health concern that constrains sustained exercise participation, reduces adherence, and compromises quality of life [16,17,18,19]. Clinically, EIMD manifests as delayed onset muscle soreness (DOMS), reductions in muscle strength, and impaired functional performance, with symptoms persisting for days to weeks [1,2,17,18]. Serum biomarkers such as creatine kinase (CK) and lactate dehydrogenase (LDH) are commonly employed to reflect sarcolemmal disruption and muscle fiber damage [20,21]. When recovery is inadequate or injury is repetitive, EIMD may progress toward chronic inflammation, fibrosis, and diminished regenerative capacity [6,8,22,23]. Importantly, the molecular pathways activated during EIMD—including inflammatory signaling, oxidative stress, and mitochondrial dysfunction—overlap substantially with those implicated in sarcopenia, metabolic dysregulation, and immunosenescence [13,19,20,22,24], positioning EIMD as a valuable experimental model for investigating skeletal muscle health and chronic disease mechanisms.

Recent advances in high-throughput sequencing and mass spectrometry have significantly propelled the application of multi-omics strategies in skeletal muscle research, expanding our understanding of the molecular responses and adaptive mechanisms of muscle under exercise-induced stress from a systems-level perspective. By integrating transcriptomics, proteomics, metabolomics, and epigenomics data, researchers have gradually revealed the dynamic remodeling of the molecular regulatory networks induced by exercise, identifying a range of potential key regulatory factors and signaling pathways. These findings provide a novel perspective on the molecular basis of structural and ultrastructural muscle damage in EIMD. For instance, transcriptomic studies indicate that factors such as training status, nutritional background, and age significantly reshape muscle gene expression profiles. Meanwhile, proteomic and phosphoproteomic analyses have further identified core molecular nodes involved in the regulation of contractile proteins, stress signaling, and the maintenance of metabolic homeostasis [25]. Building on these insights, systems biology and multi-omics frameworks have gradually elucidated several interconnected regulatory modules in EIMD, including inflammation kinetics, mitochondrial homeostasis, iron-dependent lipid peroxidation, and satellite cell fate decisions [24,26,27,28,29,30,31,32,33]. These processes should not merely be viewed as the “causes” or “results” of EIMD, but rather as stage-dependent system state transitions. In the early stages of damage, these modules typically appear as secondary responses to mechanical and metabolic stress, but when activation persists or regulation becomes unbalanced, they can drive muscle evolution from adaptive, reversible damage to maladaptive remodeling and regeneration failure. This progression is closely linked to chronic muscle dysfunction, sarcopenia related to aging, and other pathological processes. Based on the above advances, this review, from a systems biology perspective, integrates animal experiments, clinical studies, and multi-omics evidence to summarize the key pathological nodes in EIMD, as well as their multi-level stress integration imbalances [14,29,34,35,36,37,38,39,40]. The review also explores the potential applications of multi-omics in identifying individual susceptibility and precision rehabilitation [6,16,18,34,35], as well as the effectiveness and limitations of various intervention strategies in alleviating symptoms and restoring muscle homeostasis [41,42].

## 2. Methods

This integrative narrative review explores the mechanisms, pathological progression, and intervention strategies associated with EIMD from a systems biology perspective. The review synthesizes evidence from mechanistic studies, animal models, population-based observations, clinical trials, and multi-omics data, aiming to construct a regulatory framework that elucidates the initiation of EIMD by mechanical stress, its propagation through calcium dysregulation, oxidative stress, and inflammatory amplification, and its eventual resolution through repair or adaptive remodeling. A comprehensive literature search was conducted in databases including PubMed and Web of Science, covering English-language publications. Core search terms included “exercise-induced muscle damage,” “skeletal muscle injury,” “muscle regeneration,” “satellite cells,” “inflammation,” and “delayed onset muscle soreness (DOMS).” Studies relevant to EIMD mechanisms or interventions were selected, and transcriptomic data from the GEO database were analyzed for differentially expressed genes (DEGs) with a fold change > 0.5 and *p* < 0.05. Differential expression analyses were performed in R (version 4.3.3). No new experimental data were generated; all figures were derived from existing datasets. Rather than aiming for quantitative causal inference, this review emphasizes the network characteristics and regulatory logic of EIMD as a plastic stress integration system, providing a systems-level theoretical framework to inform future mechanistic validation and precision intervention research.

## 3. Mechanisms and Cellular Responses Underlying Exercise-Induced Skeletal Muscle Damage

### 3.1. Initial Structural Disruption and Calcium Homeostasis Dysregulation

**Figure 1:**
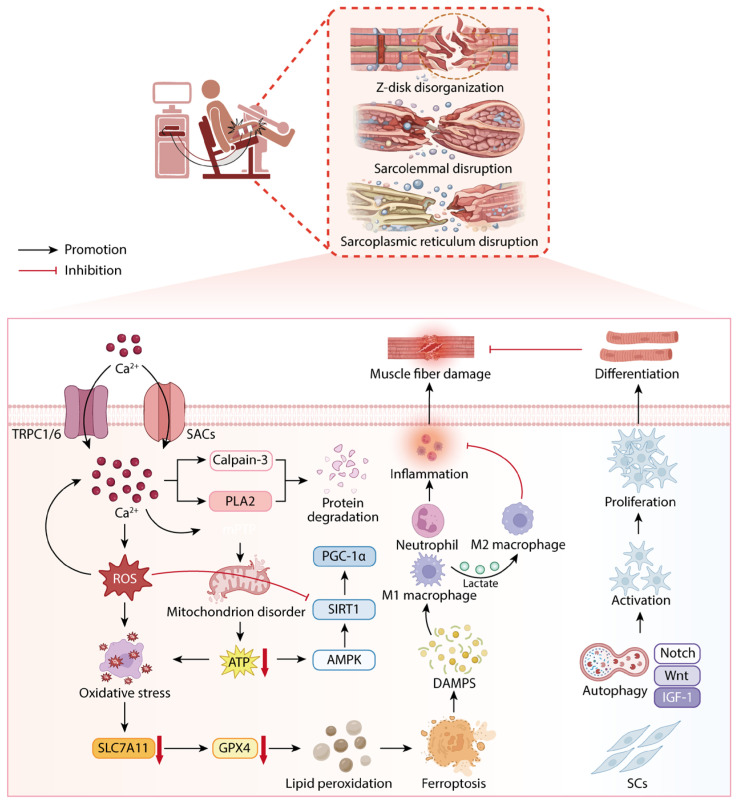
Schematic overview of the mechanisms underlying EIMD. High-intensity or unaccustomed exercise stimuli, particularly eccentric contractions, induce excessive sarcomere stretching and structural disruption of skeletal muscle fibers, manifested by Z-disk disorganization and microlesions of the sarcolemma and sarcoplasmic reticulum. These structural insults trigger intracellular Ca^2+^ homeostasis dysregulation mediated by stretch-activated channels (SACs) and TRPC1/6 channels, establishing a self-amplifying ROS–Ca^2+^ positive feedback loop. Sustained Ca^2+^ overload subsequently activates Ca^2+^-dependent effectors, including calpain-3 and phospholipase A_2_ (PLA_2_), as well as mitochondrial permeability transition pore (mPTP) opening, leading to degradation of structural proteins and mitochondrial dysfunction. Excessive oxidative stress and ferroptosis further exacerbate muscle injury through lipid peroxidation and downregulation of GPX4 and SLC7A11. Damaged myofibers release damage-associated molecular patterns (DAMPs), initiating inflammatory responses characterized by infiltration of neutrophils and M1 macrophages, which clear necrotic tissue but may cause collateral damage to surrounding fibers when excessively activated. In contrast, M2 macrophages and metabolic cues such as lactate promote inflammation resolution, tissue repair, and regeneration. Mitochondrial dysfunction results in impaired energy supply and enhanced oxidative stress, accompanied by suppression of the AMPK–SIRT1–PGC-1α signaling axis and reduced metabolic buffering capacity. Ultimately, satellite cells (SCs) contribute to myofiber repair through activation, proliferation, and differentiation, processes tightly regulated by metabolic state, key signaling pathways (Notch, Wnt, IGF-1), and the autophagy system, thereby determining long-term skeletal muscle adaptability and regenerative quality.

EIMD typically occurs following high-intensity or unaccustomed exercise, particularly with eccentric contractions as a typical trigger (Figure 1) [43,44]. During this process, skeletal muscles undergo passive stretching while actively contracting, leading to significant tension imbalance between sarcomeres, with some “weaker sarcomeres” preferentially experiencing excessive extension and disruption [7,45]. This “weaker sarcomere instability” model has been systematically regarded as the core initial event in eccentric contraction-induced muscle damage, which is fundamentally caused by excessive sarcomere strain, rather than overall muscle force levels [46]. From an ultrastructural perspective, EIMD is typically characterized by Z-disk displacement and misalignment, disordered myofibril arrangement, and stretching or scattering at the A-band and Z-disk interface. These structural changes have been confirmed through electron microscopy observations [47]. Further animal experiments have shown that even a single stretch stimulus, in an actively contracting state, can induce significantly more muscle fiber damage than under passive stretch conditions, suggesting that the active strain state plays a decisive role in the initiation of damage [48]. Additionally, ultrastructural damage includes the repositioning and structural disruption of the triad and transverse tubule system. Classic eccentric exercise models show longitudinal extension of the T-tubule system, changes in the direction of the triad, and abnormal T-tubule–terminal cisternae contact structures (e.g., “five-link/seven-link triads”), indicating that excitation-contraction coupling (E-C coupling) components are affected by mechanical stress [49,50]. This initial structural instability manifests not only as Z-disk disorganization and disruption of myofibrillar alignment but also as microlesions within the sarcolemma and transverse tubule (T-tubule) system, thereby compromising myofiber integrity and providing a physical substrate for the propagation of intracellular stress signals. Disruption of sarcolemmal and sarcoplasmic reticulum (SR) integrity directly perturbs intracellular calcium (Ca^2+^) homeostasis. Large amounts of extracellular Ca^2+^ enter the cytosol along concentration gradients, while aberrant Ca^2+^ release from SR stores further exacerbates sustained intracellular Ca^2+^ overload [45]. Beyond passive leakage, mechanical stretch activates stretch-activated channels (SACs) and transient receptor potential canonical (TRPC) channels, amplifying Ca^2+^ influx [51,52]. Among these, TRPC1 and TRPC6 are markedly upregulated in EIMD models and are considered critical molecular nodes sustaining aberrant Ca^2+^ signaling [53,54]. Importantly, Ca^2+^ dysregulation does not occur in isolation but is tightly coupled with oxidative stress. Reactive oxygen species (ROS) generated by dysfunctional mitochondria and infiltrating inflammatory cells can oxidatively modify key cysteine residues within TRPC channels (e.g., Cys553/Cys558 in TRPC5), prolonging channel open probability and thereby establishing a self-amplifying “ROS–Ca^2+^–ROS” feedback loop [55,56,57]. This positive feedback mechanism enables localized mechanical injury to rapidly escalate into cell-wide stress dysregulation and constitutes a major pathological basis for the persistence of DOMS. Sustained Ca^2+^ overload subsequently activates multiple Ca^2+^-dependent effector pathways. Aberrant activation of calpains, particularly calpain-3, promotes degradation of essential structural proteins such as titin, desmin, and dystrophin-associated complexes, thereby further destabilizing myofibrillar architecture [20,51]. Concurrently, activation of phospholipase A_2_ (PLA_2_) hydrolyzes membrane phospholipids, generating arachidonic acid and enhancing pro-inflammatory prostaglandin synthesis, which exacerbates membrane disruption. Mitochondrial Ca^2+^ overload additionally induces opening of the mitochondrial permeability transition pore (mPTP), suppresses oxidative phosphorylation, and triggers the release of pro-apoptotic mediators [51,54]. Collectively, Ca^2+^ homeostasis dysregulation represents not merely a downstream consequence of EIMD, but a central signaling hub linking mechanical stress, metabolic imbalance, and inflammatory amplification, marking a critical transition from adaptive stress responses to systemic functional instability.

### 3.2. Oxidative Stress and Ferroptosis

In the early phase of EIMD, mechanical stress and Ca^2+^ dysregulation rapidly disrupt intracellular redox homeostasis, leading to excessive accumulation of reactive oxygen species (ROS) within damaged muscle regions. ROS generation arises from multiple sources, including electron leakage from a compromised mitochondrial electron transport chain and NADPH oxidase–mediated respiratory bursts during inflammatory cell infiltration [3]. Within physiological adaptive limits, transient ROS elevations serve as signaling molecules that activate antioxidant defenses and repair pathways. However, under EIMD conditions, sustained and excessive ROS production overwhelms the cellular antioxidant buffering capacity, driving injury signals from localized to systemic propagation. ROS exert deleterious effects through lipid peroxidation, protein carbonylation, and oxidative DNA modifications, broadly impairing cellular structure and function. In parallel, ROS activate redox-sensitive transcription factors such as NF-κB, inducing sustained expression of pro-inflammatory cytokines including IL-6 and TNF-α, thereby further amplifying local inflammatory responses [40,58]. In this context, oxidative stress is no longer a mere byproduct of tissue injury but functions as a critical amplifier coupling metabolic disruption to inflammatory expansion.

Emerging evidence implicates ferroptosis as a pivotal threshold mechanism underlying oxidative stress dysregulation in EIMD [29,36,37]. Ferroptosis is a regulated form of cell death characterized by iron-dependent lipid peroxidation, which is contingent upon iron homeostasis disruption, glutathione depletion, and reduced activity of glutathione peroxidase 4 (GPX4). Under strenuous exercise conditions, iron metabolic imbalance combined with antioxidant exhaustion accelerates lipid peroxidation, further compromising mitochondrial membrane integrity and establishing a positive feedback loop of persistent ROS generation. Notably, key ferroptosis regulators such as GPX4 and SLC7A11 are significantly downregulated in EIMD models, whereas natural antioxidants (e.g., gallic acid) attenuate myofiber damage by enhancing autophagic flux and antioxidant defenses, thereby suppressing ferroptosis activation [29,36,37]. Furthermore, the extent of ferroptosis activation may correlate with exercise intensity, suggesting that it could serve as a key molecular threshold to distinguish between "reversible adaptive damage" and "irreversible functional impairment". Thus, ferroptosis should not be regarded merely as a downstream phenotype resulting from uncontrolled oxidative stress, but rather as a threshold-regulated damage amplifier capable of converting transient redox imbalance into persistent mitochondrial dysfunction and muscle fiber degeneration.

### 3.3. Inflammatory Responses and Immune Cell Polarization

Following EIMD, damaged myofibers release large quantities of damage-associated molecular patterns (DAMPs), rapidly activating the innate immune system and initiating a coordinated cascade of inflammatory cell recruitment and functional differentiation. Neutrophils are among the first responders, infiltrating injured tissue within hours of damage onset. Through respiratory bursts, neutrophils release ROS and proteolytic enzymes that facilitate clearance of necrotic debris; however, their nonspecific cytotoxic activity may also inflict collateral damage on adjacent intact myofibers [51,54]. Thus, neutrophils constitute both essential mediators of early debris clearance and potential contributors to injury amplification. Subsequently, macrophages derived from infiltrating monocytes become the dominant immune cell population. During the early inflammatory phase, classically activated M1 macrophages predominate, activating NF-κB and AP-1 signaling pathways and secreting pro-inflammatory cytokines such as TNF-α and IL-1β, thereby reinforcing the inflammatory milieu within the lesion site [20,59]. While this pro-inflammatory phase is indispensable for effective clearance of necrotic tissue, prolonged M1 dominance disrupts the regenerative microenvironment and delays repair.

As recovery progresses, macrophage phenotypes must transition toward alternatively activated M2 states, which secrete IL-10, TGF-β, and IGF-1 to promote inflammation resolution, angiogenesis, and satellite cell activation [20]. In this context, “immune mismatch” does not simply refer to the presence of inflammation, but specifically to the temporal and phenotypic misalignment between pro-inflammatory (M1-like) and pro-regenerative (M2-like) immune programs. This misalignment directly affects the direction of damage repair. In other words, immune regulation in EIMD is not only dependent on the intensity of inflammation but also highly reliant on the temporal coordination of immune cell polarization. Abnormal or delayed transitions in immune polarization are a key mechanism underlying chronic damage and impaired regeneration. It is noteworthy that metabolic status plays an important bridging role in immune regulation. Lactate, traditionally regarded as a metabolic byproduct, has been shown to promote M2 macrophage polarization via activation of receptors such as GPR81, highlighting the intimate coupling between metabolic signaling and immune decision-making [60,61,62]. In addition, heat shock proteins (HSPs), including HSP70 and HSP25, exert cytoprotective effects during EIMD. HSP70 suppresses oxidative stress-induced apoptosis mediated by the JNK/p38 MAPK pathways, whereas HSP25 stabilizes myofiber structure and reduces the risk of secondary rupture [29,37,59]. At the systems level, inflammatory responses constitute a critical “decision node” in the injury–repair transition, and dysregulated immune polarization represents a major mechanism underlying chronic injury and impaired regeneration.

### 3.4. Energy Metabolic Disturbance and Mitochondrial Dysfunction

Efficient muscle repair is critically dependent on an adequate and sustained energy supply, positioning mitochondrial function at the core of EIMD pathophysiology. Under damage-inducing exercise conditions, mitochondria function not only as executors of energy metabolism but also as integrative hubs coordinating mechanical stress, Ca^2+^ signaling, and oxidative stress. During EIMD, mitochondrial structure and function are broadly compromised, characterized by reduced activity of electron transport chain complexes I and III, diminished ATP synthesis efficiency, and markedly increased ROS leakage [63,64,65,66]. These alterations establish a vicious cycle in which energy deficiency and oxidative stress mutually reinforce one another. Ca^2+^ dysregulation represents a major upstream driver of mitochondrial dysfunction. Excessive Ca^2+^ influx into the mitochondrial matrix induces opening of the mitochondrial permeability transition pore (mPTP), leading to loss of membrane potential, interruption of oxidative phosphorylation, and release of pro-apoptotic factors such as cytochrome c, thereby activating downstream effectors including caspase-3 [51,54]. This transition marks a critical shift in which mitochondria evolve from stress buffers into amplifiers of damage signals, delineating the boundary between reversible injury and cell death. At the regulatory level, the AMPK–SIRT1–PGC-1α metabolic axis is essential for maintaining mitochondrial biogenesis and functional homeostasis. However, under EIMD conditions, activation of this axis is constrained, resulting in impaired energy sensing and insufficient mitochondrial renewal [63]. Moreover, exogenous factors such as statin therapy can further suppress this pathway, exacerbating metabolic imbalance and delaying functional recovery [66]. Thus, from a systems biology standpoint, mitochondrial dysfunction in EIMD reflects not merely an energy deficit but a convergence point of multilevel stress integration failure.

### 3.5. Regenerative Mechanisms and Stem Cell Regulatory Networks

Despite extensive structural and functional disruption induced by EIMD, skeletal muscle retains a remarkable regenerative capacity, which is primarily dependent on the activation, expansion, and fate determination of satellite cells (SCs). Residing between the basal lamina and sarcolemma, SCs maintain a quiescent, low-metabolic, undifferentiated state under homeostatic conditions. In response to injury and inflammatory cues, SCs become activated, enter proliferative and migratory programs, and differentiate to repair damaged myofibers or generate new myonuclei [67]. Metabolic state plays a decisive role in sustaining SC function. Endurance exercise has been shown to enhance SC stemness and long-term reserve capacity by modulating mitochondrial oxygen consumption rates and metabolic flexibility [68]. At the molecular level, signaling pathways including Notch, Wnt, and IGF-1/PI3K/Akt coordinately regulate SC fate decisions. IGF-1 not only promotes myofiber hypertrophy but also supports SC function by remodeling the regenerative niche [69]. In contrast, myostatin (MSTN) negatively regulates muscle growth by suppressing oxidative phosphorylation (OXPHOS), and its aberrant activation compromises regenerative quality and accelerates functional decline [63,64]. Autophagy is likewise indispensable for SC homeostasis. Deficiency of the autophagy-initiating kinase Ulk1 impairs mitochondrial turnover, promotes accumulation of metabolic waste, and suppresses SC proliferative capacity. Studies employing Pax7-CreERT2-mediated SC-specific ablation in Ulk1-deficient backgrounds further demonstrate that compromised autophagic function markedly reduces muscle regenerative potential [70,71,72,73,74,75]. Accordingly, from a systems biology perspective, SCs function not merely as executors of repair but as long-term “recorders” of cumulative stress history. Recurrent or excessive EIMD may reshape SC fate through metabolic and epigenetic regulation, thereby exerting lasting effects on muscle adaptability and overall health. Although animal models have clearly demonstrated that satellite cells play a core role in muscle regeneration and metabolic/epigenetic memory [76,77,78], direct evidence of clinical manipulation of satellite cell epigenetic memory remains limited [79]. However, human training–de-training–retraining studies have shown that muscle-specific transcriptomic and epigenetic signatures can be long-lasting [76,79,80], which aligns with the “muscle memory” hypothesis mediated by satellite cells [81]. These observations suggest that in clinical and exercise practices, strategies such as early training, metabolic conditioning, and timely resolution of inflammation can optimize the metabolic and epigenetic states of satellite cells, thereby enhancing muscle resilience when facing future EIMD [76,81].

## 4. Systems Biology Value of Animal Models in EIMD Mechanistic Research

### 4.1. Commonly Used Model Designs and Experimental Approaches

Animal models provide indispensable systems-level value in elucidating the mechanisms of EIMD, particularly for decoding how eccentric mechanical stress is translated into molecular and cellular instability. Compared with human studies, animal models allow precise control of exercise load, frequency, and injury modality while enabling temporal and genetic interventions that are critical for identifying key transition points in the progression from acute stress to chronic functional remodeling.

Currently, Sprague–Dawley (SD) rats and various genetically modified mouse strains constitute the most widely used experimental platforms in EIMD research. Standardized eccentric exercise protocols, such as downhill treadmill running and high-intensity or overtraining paradigms, are employed to recapitulate human exercise-related muscle damage. Injury severity is quantitatively assessed using histopathological analysis, serum biochemical markers (e.g., creatine kinase and lactate dehydrogenase), and inflammatory mediators including IL-6 and TNF-α [29]. From a systems biology perspective, the principal advantage of these models lies in their ability to reproduce not only damage phenotypes, but also the entire causal chain linking mechanical stress, metabolic disturbance, and inflammatory amplification.

(1) Eccentric exercise–induced injury models

SD rat models are extensively used to investigate both acute and chronic pathological features of EIMD due to their moderate body size and stable physiological responses. By adjusting treadmill incline, running speed, and training duration, injury models of varying intensity and persistence levels can be established. In addition, acute injury models induced by fixed kinetic energy (e.g., 10 J) effectively mimic early post-exercise inflammatory responses and extracellular matrix (ECM) remodeling processes [82,83]. These paradigms provide a foundational framework for studying injury amplification, inflammation persistence, and repair failure.

(2) Transgenic mouse models

To delineate causal roles of specific molecules in EIMD, transgenic and conditional knockout mouse models are widely employed. For instance, skeletal muscle-specific ALDH2 overexpression models subjected to exhaustive exercise have been used to investigate the regulatory roles of oxidative stress and mitochondrial dysfunction in exercise-induced muscle injury [84]. The value of such models lies in their capacity to transform genetic background into a variable that modulates stress-response thresholds, thereby revealing molecular determinants of inter-individual susceptibility.

(3) Disease–exercise interaction models

Skeletal muscle-specific DDAH1 knockout mice combined with swimming training and cardiotoxin (CTX) injection offer a unique platform for investigating the protective mechanisms of exercise interventions under disease conditions [85]. This model underscores that EIMD is not an isolated event; rather, its outcomes are strongly dependent on baseline metabolic status and vascular/nitric oxide signaling.

(4) Model extensions and limitations

Animal models have also been applied to simulate age-related sarcopenia and various disease-associated muscle injury states, facilitating investigation of protein metabolism imbalance, oxidative stress, and regenerative decline [86]. Nevertheless, differences in locomotor patterns, muscle fiber composition, and recovery kinetics between small animals and humans remain substantial [87,88]. Consequently, contemporary model development increasingly emphasizes systematic standardization of induction protocols and outcome measures to enhance reproducibility and translational relevance [29,89].

### 4.2. Key Mechanistic Insights Derived from Animal Models

Based on the aforementioned model systems, extensive research has identified multiple molecular pathways that play integrative roles in EIMD onset, repair, and long-term remodeling. These findings not only deepen mechanistic understanding of muscle injury physiology but also provide clear directions for targeted intervention strategies.

(1) ALDH2: A mitochondrial quality control regulator and oxidative stress buffer

Studies in ALDH2 transgenic mice demonstrate that exhaustive exercise-induced skeletal muscle damage is markedly attenuated by ALDH2 overexpression [84]. This protective effect extends beyond simple antioxidant activity and is primarily mediated through modulation of mitochondrial homeostasis, reduction in ROS accumulation, and enhancement of endogenous antioxidant enzyme systems. Specifically, ALDH2 suppresses abnormal accumulation of lipid peroxidation products such as 4-hydroxynonenal, improves exercise endurance, and reduces fatigue-associated injury risk, highlighting its central role in integrating metabolism, oxidative stress, and structural stability.

(2) DDAH1: A signal amplification node for aerobic training-induced protection

In DDAH1-deficient models, the protective effects of aerobic exercise against CTX-induced muscle injury are significantly blunted, whereas wild-type mice exhibit enhanced antioxidant capacity and regenerative potential [85]. Mechanistic analyses indicate that DDAH1 modulates nitric oxide signaling pathways to coordinately suppress inflammation and oxidative damage. These findings emphasize that the protective benefits of aerobic training are not solely attributable to mechanical stimuli but are systemically amplified through specific metabolic–vascular signaling axes.

(3) Ythdf1: A post-transcriptional “brake” on endurance exercise-induced muscle remodeling

Ythdf1-deficient mouse models have revealed a critical molecular mechanism underlying endurance exercise adaptation [90]. Endurance training suppresses Ythdf1 expression, and its deletion phenocopies exercise-induced effects, including muscle hypertrophy, increased mitochondrial content, and a higher proportion of type I fibers. Mechanistically, Ythdf1 negatively regulates translation of myostatin (Mstn), thereby indirectly relieving suppression of satellite cell activation and muscle growth. This work represents the first integration of RNA modification reader proteins into the regulatory framework of EIMD adaptation, offering novel insights into exercise-mediated improvements in muscle quality and anti-aging processes.

Overall, animal model-based studies highlight the protective roles of antioxidant defenses, metabolic signaling, and stem cell activation in EIMD while revealing complex interactions between genetic background and environmental stimuli. These insights provide a robust mechanistic foundation for the development of precision intervention strategies targeting exercise-induced skeletal muscle injury and maladaptation [85,90,91].

## 5. Systems-Level Integration of Multi-Omics Data in Elucidating EIMD Mechanisms

High-intensity or unaccustomed exercise, particularly involving eccentric contractions, induces microstructural skeletal muscle damage, manifested by characteristic features such as strength loss, DOMS, and elevated serum CK levels [92]. However, EIMD is not a singular event; rather, it constitutes a highly dynamic process encompassing mechanical disruption, immune activation, satellite cell engagement, and extracellular matrix (ECM) remodeling. Traditional single-gene or single-protein-focused approaches are insufficient to capture this process in its entirety. The advent of high-throughput sequencing and mass spectrometry technologies has enabled multi-omics strategies, providing a novel systems biology perspective on EIMD. By integrating genomic, transcriptomic, proteomic, metabolomic, and epigenomic datasets, researchers can reconstruct a comprehensive molecular cascade from genetic blueprint to phenotypic output, thereby revealing the regulatory networks and key nodes underlying EIMD [93].

### 5.1. Genomics and Transcriptomics

At the genomic level, research has focused on how inter-individual genetic variation modulates susceptibility to EIMD. For example, analysis of 20 candidate single-nucleotide polymorphisms (SNPs) demonstrated that carriers of non-dominant alleles exhibit more pronounced reductions in muscle strength and elevated DOMS following exercise [94], suggesting that polygenic risk spectra may determine individual stress thresholds. Transcriptomics, particularly RNA sequencing (RNA-seq), has become a core tool for elucidating the temporal dynamics of skeletal muscle gene expression in response to exercise. Recent studies highlight the regulatory role of RNA modifications in damage responses. Exercise-induced N6-methyladenosine (m6A) modifications can influence the stability and translational efficiency of damage-related mRNAs, while the m6A reader protein YTHDF1 plays a critical role in modulating mitochondrial function, muscle remodeling, and regenerative pathways [90,95,96,97]. Loss-of-function YTHDF1 models display endurance training-like adaptive phenotypes, underscoring the importance of post-transcriptional regulation in the metabolic reprogramming underlying EIMD. Single-cell RNA sequencing (scRNA-seq) further reveals cellular heterogeneity within the injured microenvironment. Post-exercise, Pax7^+^ satellite cells can differentiate into diverse myogenic lineages, while the interaction networks among immune cells, fibroblasts, and endothelial cells are systematically mapped [93,98,99], providing a detailed atlas of the regenerative niche.

**Figure 2:**
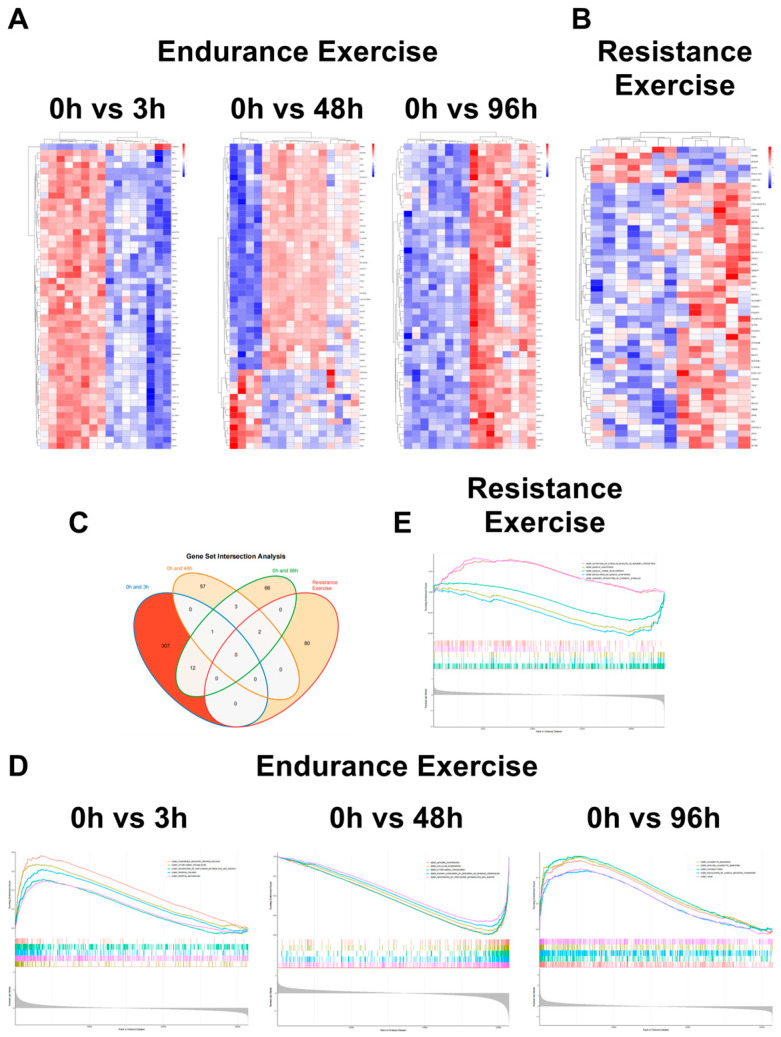
Transcriptomic profiling of skeletal muscle damage induced by different training modalities. (**A**,**B**) Heatmaps showing the top 50 significantly differentially expressed genes (DEGs) identified under two exercise conditions: endurance training at 3 h, 48 h, and 96 h post-exercise (**A**), and resistance training at 24 h post-exercise (**B**). Each column represents an individual sample, and each row represents a DEG. Color gradient from blue (downregulation) to red (upregulation) indicates normalized gene expression levels (Z-scores). (**C**) Venn diagram illustrating the overlap of DEGs in endurance training (3 h, 48 h, and 96 h) and resistance training (24 h). Minimal gene overlap was observed among conditions, indicating high modality-specific transcriptional responses and distinct regulatory mechanisms activated by different training modalities. (**D**,**E**) Gene Set Enrichment Analysis (GSEA) results of global gene expression profiles following endurance training (**D**) and resistance training (**E**). Transcriptomic datasets were obtained from the GEO database (accession numbers: GSE43856 and GSE45426). Differential expression analysis was performed using the limma package (version 3.62.2) by comparing exercise intervention groups (endurance or resistance) with corresponding control groups. Genes with log_2_-transformed fold change > 0.5 and *p* < 0.05 were considered significantly differentially expressed. Pathway significance in panels D and E was evaluated using normalized enrichment scores (NESs) and false discovery rates (FDR q-values).

To systematically compare EIMD responses under different training modalities, we curated two high-quality transcriptomic datasets representing endurance and resistance training-induced muscle damage [100,101]. Both studies involved healthy male volunteers, utilized time-series designs, and obtained high-confidence data from vastus lateralis biopsies, providing a robust foundation for investigating early transcriptional events. Neubauer et al. applied a standardized high-intensity endurance regimen (cycling plus running) and profiled skeletal muscle transcriptomes at 3, 48, and 96 h post-exercise, revealing temporal dynamics in oxidative phosphorylation, inflammatory signaling, and muscle regeneration. Murton et al., focusing on resistance training novices, captured early inflammatory and repair-related gene expression fluctuations at 24 h post-exercise. Systematic comparison of these datasets revealed pronounced modality- and time-dependent transcriptional responses. Heatmap analyses (Figure 2A,B) showed that endurance exercise elicited early activation of stress and inflammatory genes at 3 h, upregulation of ECM remodeling and immune-regulatory genes at 48 h, and stabilization of repair-related gene expression by 96 h. In contrast, resistance training induced a concentrated and robust activation of inflammatory and tissue repair genes at 24 h. Venn diagram analysis (Figure 2C) indicated limited overlap in differentially expressed genes (DEGs) between the two modalities, suggesting distinct molecular mechanisms underlying EIMD responses. Functional enrichment analysis (Figure 2D,E) further corroborated these differences: endurance exercise predominantly activated pathways associated with mitochondrial function, oxidative phosphorylation, and metabolic adaptation, whereas resistance training enriched for cytoskeletal remodeling, ECM organization, and inflammation-mediated signaling.

**Figure 3:**
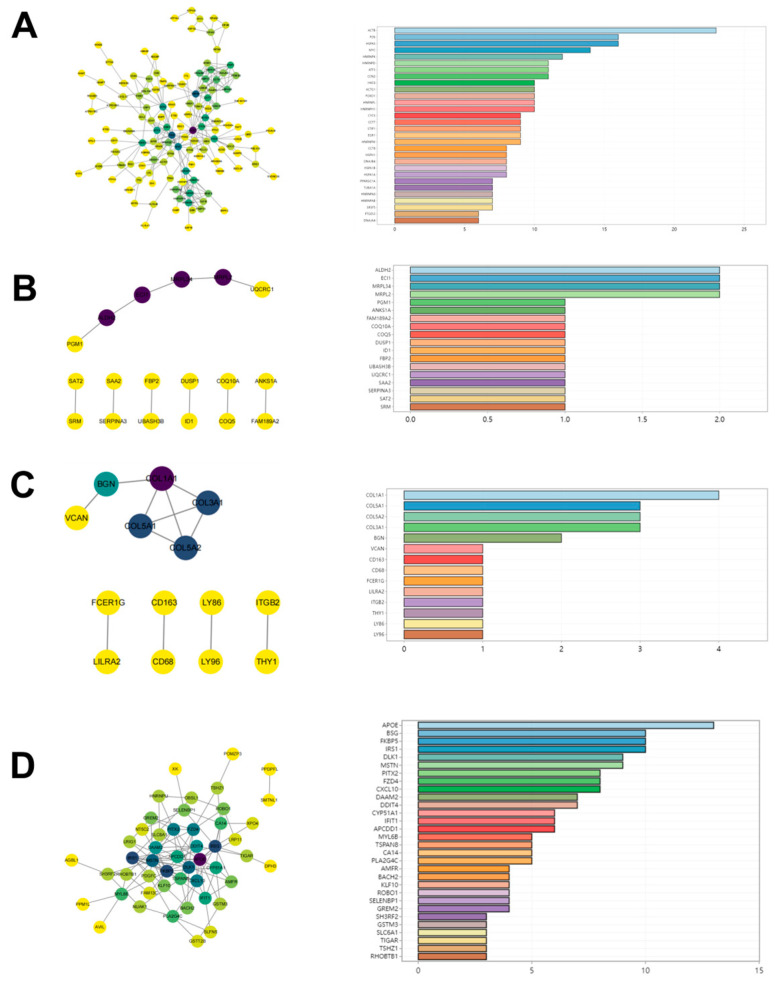
Protein–protein interaction (PPI) network analysis of differentially expressed genes following exercise-induced skeletal muscle damage. Protein–protein interaction networks were constructed for endurance training at 3 h (**A**), 48 h (**B**), and 96 h (**C**), and for resistance training at 24 h (**D**). In the PPI networks (left panels), nodes represent proteins and edges indicate protein–protein interactions. Highly connected nodes (hub genes) are characterized by high degree centrality and frequent interactions, suggesting key roles in maintaining network stability and functional coordination. The right panels display the top 30 genes ranked by node degree. PPI networks were generated using the STRING database using a confidence score > 0.7, followed by network reconstruction and visualization in Cytoscape (version 3.6.1).

To further dissect the functional implications of DEGs induced by different exercise modalities, we constructed protein–protein interaction (PPI) networks to systematically map EIMD-specific signaling pathways and functional modules. PPI networks serve as an integrative bridge in multi-omics analyses, revealing interactions among key regulatory proteins and identifying core nodes driving repair processes. Under endurance training, PPI networks exhibited pronounced time-dependent dynamics: at 3 h (Figure 3A) and 48 h (Figure 3B): core networks were enriched for redox regulation, stress response, and anti-inflammatory pathways, indicating early activation of protective mechanisms; by 96 h (Figure 3C), networks shifted toward pathways associated with tissue reconstruction and regeneration, reflecting progression toward repair stabilization. Conversely, resistance training-induced networks displayed distinct regulatory patterns. At 24 h (Figure 3D), PPI networks exhibited highly concentrated interactions enriched in ECM remodeling, muscle structural protein regulation, and immune signaling, suggesting that resistance training predominantly triggers structural stress and early tissue responses, potentially accelerating repair initiation. Cytoscape analysis identified 30 high-degree hub genes involved in inflammation regulation, cell migration, and ECM stability, indicating pivotal roles in coordinating injury responses. Identification of these hub genes not only elucidates core regulatory factors underlying modality-specific EIMD but also provides a molecular basis for targeted, training-specific intervention strategies. In summary, PPI network analysis reveals functional divergence and dynamic features in EIMD responses between endurance and resistance exercise: endurance exercise emphasizes oxidative stress buffering and anti-inflammatory responses, whereas resistance exercise prioritizes structural adaptation and rapid ECM remodeling. This network-level heterogeneity offers a systems biology framework for understanding modality-specific mechanisms of EIMD and informs the development of individualized exercise interventions.

### 5.2. Integration of Proteomics, Metabolomics, and Epigenomics

Proteins play a central role in the skeletal muscle stress response, acting as functional executors during the processes of EIMD and subsequent repair. Proteomics reveals that, following EIMD, the protein profiles associated with muscle fiber structure, energy metabolism, inflammation, and oxidative stress undergo significant remodeling [102,103]. Phosphoproteomics further uncovers the activation states of signaling pathways, providing molecular insights into the regulation of calcium homeostasis and contractile function [104]. Concurrently, the dynamic reorganization of the actin–myosin system, which runs parallel to structural damage, plays a crucial role in functional recovery and long-term adaptation, such as strength-induced muscle hypertrophy. By integrating transcriptomics and proteomics data, changes in the gene expression of actin and myosin can be depicted, connecting ultrastructural observations with molecular regulatory pathways, and shedding light on muscle remodeling mechanisms [25]. Additionally, metabolomics paints a further picture of dynamic energy and substrate flux, such as glycogen mobilization, lactate accumulation, and amino acid metabolism [105]. Integration of transcriptomics and metabolomics analyses suggests that mechanical and oxidative stress drive gene expression changes, triggering metabolic flux reprogramming [25,105,106,107]. Cross-omics integration reveals a “signal recognition–transcriptional activation–protein execution–metabolic support–adaptive feedback” regulatory loop [25,106,108,109,110], providing a theoretical framework for understanding the dynamic networks in EIMD. However, these findings also highlight current limitations, including sample size, causal verification, and standardization of computational methods [104,111,112,113].

Epigenetic mechanisms, acting as a bridge between environmental stimuli and gene expression, play a critical regulatory role in EIMD. Early on, resistance training can drive the demethylation of muscle-specific gene promoters, such as MYOD1 and MYF5, which correlates with rapid transcriptional upregulation [112,114,115], and can be partially retained after detraining, forming a “muscle memory” effect [116,117]. Post-transcriptional modifications like m6A methylation and YTHDF1 regulation further connect epigenetic control to protein synthesis dynamics [90]. Cross-omics analyses show that epigenetic remodeling can influence energy metabolism and mitochondrial function, indirectly supporting muscle repair [25]. With the application of time-series, spatial-omics, and functional gene editing technologies, dynamic molecular events and the Meta-interactome network in EIMD are gradually emerging, paving the way for constructing high spatiotemporal resolution four-dimensional dynamic regulatory maps and enabling precise mechanism elucidation and intervention strategies [25,106]. Thus, epigenetic memory serves not only as a molecular “record” of prior mechanical and metabolic stress but also as a crucial regulatory factor in determining future muscle regenerative capacity, occupying a key interface between injury history and long-term adaptive ability.

### 5.3. Cross-Validation of Multi-Omics Data and Muscle Fiber Ultrastructural Damage

The development of high-throughput omics technologies has advanced EIMD research from mere morphological descriptions to integrated molecular mapping. Multi-omics approaches enable the identification of molecular markers closely associated with skeletal muscle structural changes on a large scale and, through cross-validation with ultrastructural observations of myofibrils and membrane system damage using electron microscopy, provide molecular support for the mechanisms of exercise-induced damage. For example, combining proteomics with immunohistochemistry has revealed significant expression changes in Z-disk-related structural proteins, such as desmin and actin, after exercise, which closely correlate with myofibrillar disorganization observed through electron microscopy, establishing a clear link between molecular alterations and ultrastructural damage [118]. Furthermore, multi-omics data integration helps reveal key molecular pathways involved in muscle contraction, calcium homeostasis, and stress responses. These pathways exhibit dynamic changes not only through transcriptomic and proteomic analyses but are also validated through ultrastructural observations of damage to the transverse tubule/triad system [17,25]. Emerging single-cell RNA sequencing technologies have further unveiled the specific molecular responses of distinct cell populations to injury, providing a foundation for combining single-cell molecular characteristics with ultrastructural changes [119]. These cross-scale integrations offer a more solid theoretical foundation for future studies on injury repair mechanisms, personalized intervention strategies, and muscle health management.

## 6. Clinical Characteristics, Assessment, and Intervention Strategies of EIMD

### 6.1. Clinical Manifestations and Functional Assessment

To systematically evaluate the pathophysiological processes of EIMD and the efficacy of therapeutic interventions, randomized controlled trials (RCTs) are regarded as the gold standard in study design. In recent years, a growing number of RCTs have focused on both the interventional outcomes and mechanistic underpinnings of EIMD. The most prevalent clinical manifestation of EIMD is DOMS, which typically emerges 12–24 h after exercise and peaks between 24 and 72 h. DOMS is characterized by localized tenderness, swelling, restricted range of motion, reduced muscle strength, and impaired physical performance [8]. These symptoms are particularly evident in untrained individuals or during periods of altered training regimens, especially following eccentric exercises such as squatting, downhill running, or resistance training [120].

The pathogenesis of EIMD involves microstructural disruption of muscle fibers, increased nociceptor sensitivity triggered by the release of inflammatory mediators (e.g., IL-6 and TNF-α), and oxidative stress mediated by ROS [3,121]. Clinical evaluation is commonly performed using subjective assessment tools, such as the visual analog scale (VAS) [122], serum biochemical markers, including CK, lactate dehydrogenase (LDH), and aminotransferases (AST/ALT) [123,124], as well as functional measurements such as maximal voluntary isometric contraction (MVIC) and joint range of motion (ROM) [125,126]. In recent years, advanced imaging techniques, including magnetic resonance imaging (MRI), and neuromuscular assessment tools such as shear wave elastography (SWE) have been increasingly applied to identify and monitor EIMD by quantifying muscle edema and stiffness. In parallel, surface electromyography (sEMG) and wearable technologies have enabled real-time monitoring of neuromuscular activity and exercise performance [7,127].

Notably, substantial inter-individual variability exists in the clinical presentation and recovery capacity following EIMD. For example, older adults typically exhibit more pronounced functional impairment and delayed recovery after equivalent exercise stimuli, which is closely associated with age-related declines in mitochondrial function and protein synthesis capacity [128,129,130]. These findings underscore the necessity of tailoring exercise prescriptions to individual physiological states, particularly in aging populations, by incorporating longer recovery periods and lower training intensities. In addition, Mendelian randomization (MR) studies have emerged as a powerful tool for causal inference by leveraging genetic variants to minimize confounding effects. MR analyses have been applied to investigate the influence of genetic variation on muscle repair, inflammatory responses, and susceptibility to EIMD. Variants in key genes such as MYLK and ACTN3 have been shown to modulate inflammatory intensity and tissue repair rates [35,131], providing a mechanistic explanation for inter-individual differences in injury severity and recovery under comparable training loads [132].

### 6.2. Advances in Non-Pharmacological, Pharmacological, and Regenerative Interventions

A wide range of nutritional supplements have demonstrated potential protective or restorative effects against EIMD, including omega-3 fatty acids (fish oil), curcumin, capsaicin, whey protein concentrates, vitamins C and E, and polyphenol-rich fruit extracts (Table 1). High-dose omega-3 supplementation has been shown to accelerate recovery of vertical jump performance, although tolerability should be considered [133]. Curcumin and capsaicin effectively alleviate DOMS while preserving maximal voluntary contraction (MVC) and joint range of motion (ROM) [134,135]. Whey protein and other protein supplements facilitate post-exercise muscle remodeling, preserve muscle function, and reduce serum CK levels [136,137,138]. Antioxidants such as vitamins C/E, green tea, and polyphenol-rich fruit juices attenuate oxidative stress and inflammatory biomarkers; however, optimal dosing, intervention duration, and bioavailability require further optimization [139,140,141,142]. Botanical and herbal supplements (e.g., ginseng, curcumin, Brazil nuts, spirulina, and salidroside) exhibit potential benefits in improving muscle damage and inflammatory indices, although evidence is largely derived from small-scale studies, necessitating validation of long-term efficacy and dose optimization [124,142,143,144,145]. Similarly, specific dietary interventions, including milk, blackcurrant, grape, and blueberry juice, have shown modest efficacy in modulating post-exercise inflammation and oxidative stress [146,147,148,149].

Physical rehabilitation strategies—such as cold-water immersion (CWI), compression garments, heat therapy (HT), low-level laser therapy (LLLT), electroacupuncture, and neural mobilization (NM)—have demonstrated varying degrees of effectiveness in alleviating DOMS, improving muscle function, and regulating muscle damage biomarkers, but some interventions may inhibit muscle hypertrophy or are limited by the type of exercise performed [150,151,152,153,154,155,156,157,158] (Table 1). For instance, CWI may reduce inflammation in the acute phase but has been reported to attenuate muscle hypertrophy when applied chronically [151], whereas compression garments consistently reduce muscle soreness and facilitate functional recovery [154,155].

Pharmacological interventions, including nonsteroidal anti-inflammatory drugs (NSAIDs; e.g., ibuprofen), tadalafil, and cannabidiol (CBD) oil, provide short-term analgesic effects and improve certain muscle damage indicators, although concerns remain regarding potential cognitive effects and long-term safety [159,160,161] (Table 1). Other agents—such as histamine receptor antagonists, 17β-estradiol, arachidonic acid, methylsulfonylmethane (MSM), and palmitoylethanolamide (PEA)—exert limited or context-dependent effects on inflammatory regulation and antioxidant capacity [162,163,164,165,166,167].

In summary, current EIMD intervention strategies primarily include three categories: nutrition (e.g., plant bioactive substances), physical rehabilitation, and pharmacological treatments. However, the effectiveness of these interventions is influenced by various factors, such as dosage, timing of administration, duration of intervention, individual differences in subjects, and variations in study design. Most interventions can alleviate muscle damage, delayed-onset muscle soreness (DOMS), and associated inflammation in the short term, but their long-term effects and clinical translation still require further validation through high-quality, multi-center, randomized controlled trials. While nutritional, pharmacological, and regenerative medical strategies show some efficacy in alleviating EIMD at different stages, a single intervention model struggles to cover the multi-level, dynamic regulatory network of the injury-repair process. Therefore, mechanism-based combinatorial strategies (e.g., combining antioxidant and immune modulation interventions with metabolic conditioning) may better align with the system biology features of skeletal muscle repair. However, to avoid interference with adaptive signaling, the timing windows, doses, and interactions between different interventions must be carefully designed.

### 6.3. Advances in Multidimensional Biomarkers and Assessment Technologies

In recent years, assessment strategies for EIMD have expanded substantially. Conventional biochemical markers such as CK, LDH, and cardiac troponin I (cTnI) remain widely used in clinical practice; however, their diagnostic utility is limited by poor specificity and delayed peak responses [168,169]. More recently, α-actin—a structural protein of the Z-disk—has emerged as a promising early biomarker, detectable within 1 h of muscle injury and remaining elevated for up to 72 h, thereby offering superior temporal sensitivity [170,171,172]. Combined assessment of α-actin and cTnI enables more accurate differentiation between skeletal and cardiac muscle injury [169]. Nevertheless, standardized detection protocols and diagnostic thresholds are lacking, and current evidence is largely derived from small cohorts, underscoring the need for multicenter validation [169,173,174].

At the technological level, noninvasive modalities such as shear wave elastography (SWE), MR T2 mapping, and multifrequency bioelectrical impedance analysis (BIA) have demonstrated advantages in dynamically assessing muscle stiffness, edema, and cellular integrity [83,175,176]. Wearable devices now enable continuous monitoring of biomarkers such as CK and inflammatory mediators, offering potential applications in real-world exercise scenarios for early risk prediction [18]. Biomarkers associated with M1/M2 macrophage polarization (e.g., Clec10a and Mrc2), as well as multiparametric models integrating EMG, ultrasound, IL-6, TNF-α, and 4-hydroxynonenal (4-HNE), further enhance diagnostic precision [177,178,179,180,181]. In addition, standardized indices—including pressure pain threshold (PPT), MVIC decline rate, and the CK/LDH ratio—have been applied to injury severity grading and rehabilitation progress monitoring [182,183,184].

**Table 1:** Overview of intervention strategies and their effects on EIMD.

Intervention	Study Design	Dose/Parameters	Outcome Measures	Key Findings	Time Window	Limitations/Translational Considerations	Reference
Fish oil (Omega-3)	RCT	2–6 g/day EPA + DHA	CK, DOMS, vertical jump (VJ)	The 6 g/day group exhibited the most rapid recovery of vertical jump performance	48–72 h	Tolerability and safety of high doses require further validation	VanDusseldorp (2020) [133]
Curcumin	CS	180 mg/day curcumin	MVC torque, ROM, muscle soreness, serum CK, plasma IL-8	Higher MVC torque (3–7 d) and ROM (2–7 d) post-exercise, with reduced muscle soreness and CK activity (3–7 d)	Pre-exercise to 7 d post	Low bioavailability; advanced formulations (e.g., nano-curcumin) recommended	Tanabe (2019) [134]
Capsaicin	RCT	12 mg/day	VJH, PPT, TCM, isokinetic/isometric strength, DOMS	Acute supplementation attenuated DOMS and improved VJH and pressure pain threshold	48 h	Dose tolerability must be carefully assessed	Rashki, M., et al. (2025) [135]
PRO	RCT	4 × 20 g on exercise day; 20 g/day for 8 subsequent days	Muscle performance, proteasome peptidase activity	Promoted muscle remodeling and preserved function under exercise-induced inflammatory conditions	2–8 d	Individualized protein requirements should be considered	Draganidis (2017) [136]
Vitamin C + E	RCT	Not specified	Peak isometric knee flexor/extensor torque, oxidative stress and inflammatory markers	Reduced oxidative stress and inflammatory responses	6 weeks	Limited generalizability across populations and protocols	Bailey et al., (2010) [139]
Lemon verbena extract (Recoverben^®^)	RCT	400 mg/day for 10 days	Muscle strength (isokinetic dynamometry)	Significantly enhanced recovery of muscle strength	Day 10 post-supplementation	Small sample size; requires larger confirmatory trials	Buchwald-Werner, S., et al. (2018) [185]
Milk	CS	Post-exercise ingestion	Serum IL-1β, IL-6, IL-10, TNF-α	Differential IL-10 responses; reduced relative IL-1β and IL-10 inflammatory responses within 48 h	Acute post-exercise	Limited population applicability	Fraschetti, E. C., et al. 2022 [146]
Blackcurrant nectar	RCT	32 oz/day	Muscle soreness, blood biomarkers	Significantly reduced muscle damage and inflammation	8 days	Small sample size	Hutchison, A. T., et al. (2016) [147]
Ginseng supplementation	RCT	20 g/day	Serum CK, IL-6, TNF-α	Significant reductions in IL-6 and CK levels	Immediate—48 h post-exercise; up to 7 d	Small sample size	Jung, H. L., et al. (2011) [142]
Spirulina supplementation	CS	42 mg/kg/day	Serum CK, LDH	Outcomes not clearly reported	0–72 h	Small sample size	Krokidas, A., et al. (2024) [144]
Probiotics	RCT	6-week intervention	VO_2_max, exercise performance	No clear effects reported	6 weeks	Longer intervention periods may be required	Lee, M. C., et al. (2024) [186]
Grapes	Not specified	Daily grape consumption	VO_2_max, work capacity, mood, perceived health, inflammation, pain, arm function	Beneficial effects on post-exercise oxidative stress and inflammation	6 weeks	Small sample size; dose-response and dietary interactions unclear	O’Connor, P. J., et al. (2013) [148]
Coenzyme Q10	DB	200 mg/day	Serum CK, LDH, MDA, SOD, GSH-Px	Did not prevent exercise-induced muscle damage or oxidative stress	4 weeks	Limited statistical power due to small sample size	Okudan, N., et al. (2018) [149]
Salidroside	RCT	300 mg/day	Endurance, strength, recovery indices	Improved endurance performance	8 weeks	Small sample size	Schwarz, N. A., et al. (2024) [145]
Protein supplementation	DB	25 g whey protein	Serum CK, subjective soreness, fatigue	Reduced muscle damage and soreness	Not specified	Small sample size	Ten Haaf, D. S. M., et al. (2020) [137]
Polyphenol-rich berry juice	RCT	Twice daily supplementation	Muscle damage, oxidative stress, inflammatory markers, leg strength	Accelerated recovery and significant improvements in leg strength	Assessed after 6 days of intensive endurance exercise	Small sample size	Valder, S., et al. (2024) [141]
Oat protein supplementation	CE	25 g/day for 7 days	Serum CK, LDH	Significantly reduced muscle damage markers and accelerated recovery	Pre- and post-intervention; 24 h and 48 h post-exercise	Small sample size	Xia, Z., et al. (2018) [138]
810-nm LLLT	RCT	10, 30, or 50 J (200 mW, 810 nm)	MVC, DOMS, CK, IL-6	50 J dose increased MVC and reduced CK	0–96 h	Standardization of dose and wavelength required	Vanin 2016 [150]
CWI	RCT	10 °C × 10 min	Muscle function, morphology, molecular markers	Attenuated post-exercise satellite cell responses and hypertrophy-related kinase activity	24–48 h	Long-term use may impair hypertrophy	Roberts 2015 [151]
Compression socks	Sim	Worn before and after competition	EIMD indices (not specified)	Effects on EIMD markers remain inconclusive	During and post-competition	Limited efficacy	Bieuzen et al., 2014 [152]
CWI	RM	Not specified	Free testosterone, IL-6, TNF-α	Potential attenuation and delay of testosterone and cytokine elevations	Post-resistance exercise	Requires further investigation	Earp, J. E., et al. (2019) [153]
Compression garments	RM	Worn for 24 h post-exercise	Ultrasound elastography, pain scores	Reduced muscle stiffness and perceived pain	24–72 h post-exercise	Relatively small sample size	Heiss, R., et al. (2018) [154]
Tight-fit garments	RCT	Worn for 72 h post-marathon	CK, DOMS, sprint time, balance, jump height	Lower CK and DOMS; higher jump performance vs. placebo	24–48 h	Limited to endurance running	Hill, J. A., et al. (2014) [155]
Electroacupuncture	RCT	Not specified	DOMS, muscle damage and oxidative stress markers	Not reported	Not specified	Insufficient methodological detail	Komine, S., et al. (2025) [156]
MHVS	CS	Not specified	Muscle damage biomarkers	No significant effects observed	Early post-injury	Small sample size	McLoughlin, T. J., et al. (2004) [157]
NM	RCT	Not specified	Pain scores, muscle swelling, ROM	Significantly alleviated DOMS	24–72 h	Small sample; untrained male participants only	Sozlu, U., et al. (2025) [179]
HT	RCT	45 °C × 90 min/day × 5 days	Peak isokinetic strength, fatigue resistance, VEGF mRNA	Increased VEGF mRNA and Ang-1 protein expression	1–4 d	Optimization of temperature and frequency needed	Kim (2019) [158]
NSAIDs (Ibuprofen)	RCT	1200 mg/day	Neutrophils, macrophages, CK, myoglobin	No significant effects on inflammation, muscle damage, or soreness	3–24 h	Not recommended for routine use	Vella (2016) [159]
Curcumin	RCT	50 or 200 mg/day	CRP, muscle strength, body composition, swelling	Significantly reduced pain and DOMS	7 days	Optimal dose and timing remain unclear	Amalraj et al., (2020) [124]
Hydrocodone bitartrate + ibuprofen	RCT	Combination vs. ibuprofen alone	Cognitive tests, motor function, pain	Improved pain and function but impaired cognition	Up to 72 h	Cognitive side effects require caution	Allen et al., (2003) [187]
Tadalafil	RCT	10 mg/day for 3 days pre- and post-EIMD	CK, LDH, IL-6, TAC, TBARS	Reduced CK, LDH, IL-6 and enhanced antioxidant capacity	0–72 h	Small sample; further trials required	Ceci, R., et al. (2015) [160]
Cannabidiol (CBD) oil	RCT	50 mg/day for 7 days	Serum CK, pain scores, functional tests	Reduced CK, pain, and improved function	7 days	Small sample; long-term safety unknown	Cochrane-Snyman, K. C., et al. (2021) [161]
Histamine receptor antagonists	RCT	Single oral dose ~60 min pre-exercise	Blood flow, inflammation, muscle damage, oxidative stress	Slight reduction in DOMS at 72 h vs. placebo	0–72 h	Marginal benefits	Ely, M. R., et al. (2017) [162]
17β-Estradiol	RCT	1 mg	Neutrophils, IL-6, hormones, CK, TAC	Significantly reduced neutrophil infiltration	4 h post-exercise	Male participants only	MacNeil, L. G., et al. (2011) [163]
ARA	RCT	1.5 g/day for 4 weeks	IL-6, CRP	Transient enhancement of acute inflammatory response	Multiple time points	Small sample size	Markworth, J. F., et al. (2018) [164]
MSM	RCT	3 g/day	Anti-inflammatory gene expression, oxidative stress	Upregulated anti-inflammatory gene expression	8 weeks	Limited generalizability	McFarlin, B. K., et al. (2025) [165]
Diclofenac sodium	RCT	Twice daily for 27 days	CK	Significantly reduced CK levels	1 week post-treatment	Long-term safety requires evaluation	O’Grady, M., et al. (2000) [166]
PEA	RCT	300–1200 mg/day	Strength recovery, DOMS, CK	No improvement in strength, soreness, or CK	7 days	Small sample size	Schouten, M., et al. (2024) [167]
Yeast β-glucan	DB	650 mg/day	Inflammatory markers	Significant reductions in inflammatory biomarkers	4 weeks	Long-term effects unclear	Zabriskie, H. A., et al. (2020) [188]
Ginger	RCT	2 g/day	VAS, limb volume, ROM, strength, CK, PGE_2_	Reduced eccentric exercise-induced pain and PGE_2_	24–48 h or 11 days	Optimal dosing unclear	Black, C. D., et al. (2010) [189]
EMW	RCT	Daily consumption	Muscle damage biomarkers, functional recovery	Preserved muscle function and reduced CK and CRP	−7 d to +72 h	Athletic applicability requires validation	Borsa, P. A., et al. (2013) [190]
Green tea extract	RCT	~980 mg/day for 4 weeks	Antioxidant capacity, lipid peroxidation, uric acid, GSH-Px	Significantly reduced lipid peroxidation	4 weeks	Small sample size	Jówko, E., et al. (2015) [140]
Greenshell mussel powder	RCT	400 mg/day for 7 days	CK, CRP, ferritin, VAS, leg strength	Reduced CK and soreness; increased leg strength	7 days	Limited to untrained healthy males	Lomiwes, D., et al. (2023) [191]
Yerba mate	RCT	1 L/day for 11 days	MVC, MDA, SOD	Reduced MDA and increased SOD	7 days	Small sample size	Panza, V. P., et al. (2016) [192]
Blueberry juice	RCT	Not specified	CK, LDH, CRP, IL-6, TNF-α	Modulated NF-κB-related inflammatory markers	Pre- to post-exercise	Limited representativeness	Lynn, A., et al. (2018) [193]
Quercetin	RCT	1000 mg/day (2–6 weeks)	MVC, VAS, CK, cytokines, IGFs, MDA	Attenuated eccentric exercise-induced muscle damage and inflammation	Pre- and post-exercise	High heterogeneity	O’Fallon, K. S., et al. (2012) [194]
Flavanol-rich cocoa beverage	RCT	Not specified	CK, muscle tenderness	Not reported	Acute post-exercise	Insufficient data	Peschek, K., et al. (2013) [195]
Jaboticaba	RCT	250 mg/day × 7 days	Plasma GSH, Mb, DOMS, MVC, MQf, MQm	Increased GSH, reduced DOMS, accelerated recovery of muscle strength and quality	2–72 h post-exercise	Bioactive compounds remain to be identified	Junior, O., et al. (2025) [143]

Table Legend: RCT: Randomized Controlled Trial; CS: Crossover Study; DB: Double-Blind; Sim: Simulation Study; CE: Controlled Experiment; RM: Repeated Measures Study; PRO: Protein (Milk Protein Concentrate, MPC); EIMD: Exercise-Induced Muscle Damage; CK: Creatine Kinase; DOMS: Delayed Onset Muscle Soreness; VJ/VJH: Vertical Jump/Vertical Jump Height; MVC: Maximum Voluntary Contraction; ROM: Range of Motion; PPT: Pressure Pain Threshold; TCM: Thigh Circumference Measurement; LDH: Lactate Dehydrogenase; IL-1β/IL-6/IL-8/IL-10: Interleukin 1β/6/8/10; TNF-α: Tumor Necrosis Factor-alpha; VO_2_max: Maximal Oxygen Uptake; MDA: Malondialdehyde; SOD: Superoxide Dismutase; GSH/GSH-Px: Glutathione/Glutathione Peroxidase; VEGF: Vascular Endothelial Growth Factor; Ang-1: Angiopoietin-1; Mb: Myoglobin; TAC: Total Antioxidant Capacity; TBARS: Thiobarbituric Acid Reactive Substances; PEA: Palmitoylethanolamide; MSM: Methylsulfonylmethane; NSAIDs: Non-Steroidal Anti-Inflammatory Drugs; MHVS: Muscle High-Voltage Stimulation; NM: Neuromobilization; HT: Heat Therapy; CWI: Cold Water Immersion; EMW: Electrolyzed Modified Water; CRP: C-reactive Protein; VAS: Visual Analog Scale (Pain Intensity); IGFs: Insulin-like Growth Factors; MQf: Functional Muscle Quality; MQm: Morphological Muscle Quality.

## 7. Research Advances and Challenges

### 7.1. Limitations of Existing Treatment Strategies and Intervention Target Gradation

Despite advancements in EIMD intervention research, challenges remain in translating these strategies into clinical practice. Firstly, individual differences significantly affect therapeutic efficacy, with factors such as sex, age, and genetic background modulating the stress response [123]. Secondly, intervention timing has yet to be standardized, as many studies fail to distinguish between the “damage amplification phase” and the “repair initiation phase,” leading to inconsistent intervention effects [196]. Thirdly, regenerative medical approaches (e.g., PRP) lack standardized preparation methods, patient backgrounds, and long-term follow-up protocols, limiting reproducibility [15].

From a translational medicine perspective, the molecular and cellular mechanisms in EIMD exhibit significant levels of intervention potential, which can guide the design of preventive, acute, and long-term intervention strategies. Core stress nodes such as ferroptosis, mitochondrial redox homeostasis, and immune polarization not only amplify injury but can also be modulated through targeted interventions. Ferroptosis, driven by lipid peroxidation, has been validated in animal and cell experiments as a key process in skeletal muscle injury and regeneration. Targeted strategies, including antioxidant interventions, iron homeostasis regulation, and Nrf2 activation, can alleviate oxidative stress and damage, thereby mitigating exercise-induced injury and related pathological phenotypes [29,197,198]. Mitochondrial homeostasis regulation, such as PINK1/Parkin-mediated autophagy, can reduce ROS accumulation and maintain energy metabolism, also showing high intervention value [17,29]. However, currently, there are no feasible clinical interventions targeting upstream mechanical stress, and downstream epigenetic memory, such as long-term reprogramming of muscle satellite cells, is more applicable to long-term training adaptations and chronic disease risk management than to acute injury intervention [199]. Thus, constructing intervention targets centered on ferroptosis, mitochondrial function, and immune polarization can optimize precise intervention strategies in different clinical settings [17,198].

It is important to note that the distinction between adaptive and maladaptive EIMD should not be defined by a single biomarker or numerical threshold, but rather understood within a multidimensional threshold framework. Key dimensions include: (1) the duration of oxidative and inflammatory signaling activation; (2) the reversibility of mitochondrial dysfunction; (3) the recovery and renewal capacity of the satellite cell pool; and (4) whether the immune response can shift from a pro-inflammatory to a pro-regenerative state in a timely manner. When these parameters fail to return to homeostasis within the established recovery window, adaptive damage may evolve into maladaptive remodeling. Based on this multidimensional threshold model, we can more systematically understand the different damage processes in EIMD and provide a theoretical basis for selecting and optimizing clinical intervention strategies.

### 7.2. Future Research Directions

While multi-omics technologies are indispensable for deciphering the molecular networks in EIMD, clinical translation still faces challenges such as high costs, limited temporal resolution, and difficulties in causal inference. The key to future progress lies in compressing various omics data into actionable biomarker combinations, integrating them with digital phenotypic monitoring and longitudinal functional assessments to achieve mechanism-driven precise interventions and improve the applicability and generalizability of the research. Future studies should focus on three key areas: (1) High-resolution mapping of mechanisms: The central role of satellite cells in muscle repair is widely recognized, but their fate conversion, metabolic reprogramming, and dynamic interaction with immune cells under different exercise injury models remain incompletely understood. Abreu and Kowaltowski (2020) discovered that endurance training promotes satellite cell self-renewal by inhibiting mitochondrial oxygen consumption, revealing the key role of metabolic pathways in stem cell fate determination [68]. Ultrastructural verification of molecular characteristics and muscle fiber stress responses will provide a theoretical foundation for refining the EIMD mechanism map. (2) Multimodal therapeutic integration strategies: A single treatment is unlikely to cover the entire muscle damage-inflammation-repair process. Integrated strategies such as “physical intervention + biomaterials + drug release + personalized exercise prescriptions” may represent a new paradigm. Alcazar et al. (2020) demonstrated that an IGF-1 sustained-release scaffold combined with autonomous movement effectively promotes angiogenesis and neuromuscular junction reconstruction, highlighting the advantages of integrated therapy in reconstructing the muscle microenvironment [69]. (3) Digital and smart rehabilitation monitoring: The development of wearable devices, digital sensors, and AI algorithms could enable real-time, personalized monitoring of muscle activity, fatigue states, and inflammation. Alvarez et al. (2022) developed a soft strain sensor capable of continuously tracking muscle mechanical performance, providing a basis for dynamically adjusting training plans [127]. In the future, combining predictive modeling with recovery feedback will enable closed-loop, mechanism-driven precise rehabilitation management.

## 8. Conclusions and Perspectives

Collectively, current evidence indicates that EIMD is not a singular injury event but rather a dynamic stress system driven by the convergence of mechanical disruption, calcium homeostasis imbalance, oxidative stress, and immune–inflammatory cascades. Mitochondrial dysfunction and ROS accumulation serve as early amplification nodes, whereas the balance between inflammation and regeneration, together with satellite cell fate determination, governs long-term adaptive outcomes. Multi-omics studies have revealed the hierarchical regulatory architecture underlying these processes, providing a theoretical foundation for unified damage–repair models. Although current interventions are transitioning from empirical symptom management toward mechanism-oriented systemic reprogramming, critical challenges remain regarding intervention timing, individual variability, and long-term safety. Future integration of multi-omics profiling, digital monitoring, and multimodal interventions holds promise for a paradigm shift from symptom alleviation to precision repair, offering scalable clinical templates for sports rehabilitation, sarcopenia, and related chronic conditions.

## Data Availability

No new data was created in this study. Data sharing is not applicable to this article.
